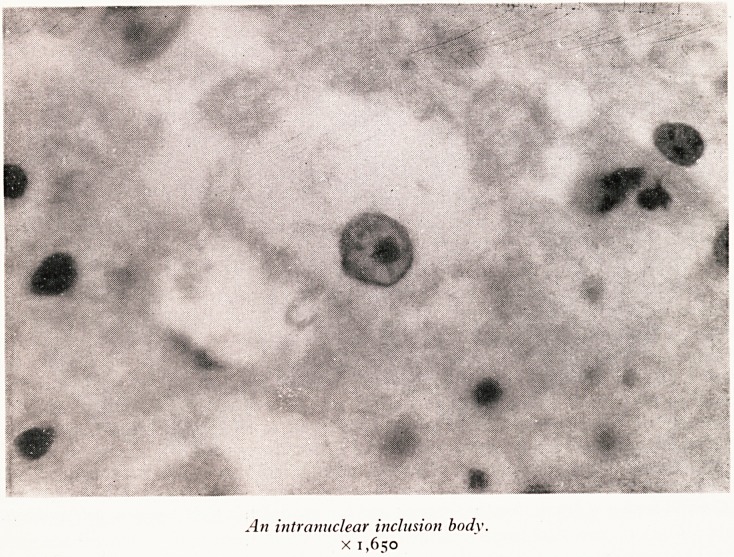# Acute Encephalitis

**Published:** 1962

**Authors:** T. F. Hewer


### ACUTE ENCEPHALITIS

*A Clinico-Pathological Conference held on the 13th February*, 1962

(P.M. No. F. 158/61)

CHAIRMAN: PROFESSOR T. F. HEWER

*Dr. J. Macrae:* Mrs. C., aged 57 years, was admitted to Ham Green Hospital under my care on 11th August last as a case of pyrexia of unknown origin. She had a history of headache and vomiting for 10 days and of a pyrexia reaching 101–102°F during the previous two days. The day before she came to hospital she was considerably confused. She had been attended by two of a partnership of general practitioners during the previous four days. The first G.P. thought she was suffering from influenza and gave her an aspirin mixture. The day after she was given some tetracycline, but this had no effect in reducing her temperature. Her husband had been treated for pulmonary tuberculosis. The patient herself had no particular relevant medical history. When we saw her first she was very confused and unco-operative and it was quite impossible to get a decent history from the patient. Her husband was with her and he gave the history that we have. He was not very clear, either, about what had been happening, and indeed he suggested that she had been vomiting off and on for a month, but he denied that later on, so we were left with a 10-day history. Her temperature on admission was 102°F., pulse 100/min; she was breathing very rapidly at 44/min. There was some neck stiffness. When we examined her we found that her fundi were normal; there was a possibility of left hemiparesis but this was very difficult to assess since she was not co-operative; similarly she might have had a left homonymous hemianopia, but again that was difficult to assess. We could not make anything at all of her sensation. We did a lumbar puncture and found that she had a pressure of 180 mm of water and there was a free rise and fall of cerebro-spinal fluid. There were 20 cells per cmm. and of these 98 per cent were lymphocytes. Protein was 45 mg/100 ml and sugar 60 mg/100 ml. Her blood sugar was 72 mg and her blood urea 27 mg per 100 ml. The white cell count was 10,700 per cmm and of these 80 per cent were polymorphs. We had a portable X-ray of her chest done and there was nothing significant in that. My initial thought about this woman was that she might be suffering from tuberculous meningitis, particularly with the history of pulmonary tuberculosis in the husband, but the cerebro-spinal fluid rather discounted that with a sugar of 60 mg/100 ml. My next thought was that she might have a brain abscess or that she might be a case of encephalitis. The possibility of brain abscess was so important that we rang up Mr. Hulme to see what he would think about it, and he suggested that we sent the case to Frenchay Hospital to the Neurosurgical Unit so that he could assess the situation properly. She was on her way to Frenchay Hospital four hours after she came in to us.

*Mr. A. Hulme:* Her condition on arrival at Frenchay was very much as Dr. Macrae has already told you. One thing which struck me very forcibly was the rather odd mental state which this lady displayed. She was quite alert but extremely confused, and although she would converse and co-operate in a rather limited way in examination, her conversation was entirely irrelevant to what one was discussing with her, and her thought content seemed to be concerned mainly with her efforts to exchange a washing machine for a small modern house. In fact she tried very hard to sell me a washing machine, but as I already had one I could not help her. Like Dr. Macrae we were not very sure about this left homonymous hemianopia. She had a left hemiparesis pretty definitely, she was not moving her left limbs as well as her right, but sensation was impossible to test with any accuracy. The next step we took was to do a right carotid arteriogram. She had been referred, as you know, as a possible case of brain abscess, and we felt that it was essential to exclude this as far as possible. Dr. Thomson will show you the X-rays.

*Dr. G. Thomson:* A right carotid angiogram was done on the patient the morning after her arrival (obviously we did the right side because she had left-sided weakness). There was no gross displacement, but in the lateral view there was stretching of the vessels, indicating some swelling of the brain. A later film did not give any further indication of the nature of the swelling; there were no pathological vessels. All we could determine was that there was a slight swelling, the sort of thing you might get with oedema or a brain abscess.

Four days later we did an air encephalogram, just to see whether there was any further swelling, or whether we could locate more accurately the swelling we had already picked up. The septum pellucidum was a little across to the left, and the right lateral ventricle appeared smaller than the left. This, of course, would also indicate some swelling of the right hemisphere.

*Mr. Hulme:* We did not feel on the strength of the arteriogram that there was sufficient evidence of a space-occupying lesion to do exploratory burr holes at that time, but on the 14th August an E.E.G. was done, which showed high amplitude very slow delta discharges, mainly focal in the right frontal region, but spreading backwards to other areas in the right hemisphere and also across the mid-line into the left frontal region. The comment of the electroencephalographer was that in view of the very slow nature of the discharges an abscess in the right frontal region could not be excluded, but the shape of the wave form was more compatible with encephalitis.

That was on the 14th August. Two days later, the day on which the air encephalogram was done, her condition had deteriorated somewhat; she had become less rousable and she had moderate neck rigidity. On that occasion a right frontal burr hole was done and also a right parietal burr hole. A needle was inserted into the frontal lobe and also into the frontal horn of the ventricle, which appeared to be full of pus, and a catheter was inserted to allow antibiotics to be instilled. We also obtained, at that time, a few small fragments of cerebral tissue which were sent to Dr. Sandry for examination. We had been treating her with tetracycline systemically, and over the course of the next few days her condition remained essentially unchanged, but by the 18th August, 7 days after admission, she had become virtually comatose, and because we were having difficulty in keeping her airway clear a tracheostomy was performed. Unfortunately she continued to deteriorate overnight and died on 19th August. I do not think any further relevant investigations were done. We did repeat the C.S.F. examination on one or two occasions, the findings being rather similar to those at Ham Green; there was a moderate increase in white cells, on one occasion to 41 per cu mm, all of them lymphocytes, with a protein of 80 mg per 100 ml. A swab of the "pus" from the frontal horn showed many leucocytes but no organisms, and nothing was grown on culture.

*Professor T. F. Hewer:* Was there sepsis in any other part of the body?

*Mr. Hulme:* No, none at all.

*Dr. R. J. Sandry:* The biopsy showed a quite severe degree of lymphocytic cuffing around a small vessel, and in the white matter there were some degenerate cells and nuclei. There was probably some oligodendroglial proliferation, and there were small groups of fat-laden phagocytic cells. There was no sign of tumour. On this very limited basis I went so unusually far as to say that I thought that this might well be an encephalitis of some sort. I dare say that I had previously made sure that this was a likely, or at least an acceptable, diagnosis!

*Question:* How much pus did you get?

*Mr. Hulme:* Two or three ccs.

*Question:* Isn't it rather odd that you got pus from the brain when the cerebrospinal fluid only contained 41 lymphocytes?

*Mr. Hulme:* Yes, that certainly is rather odd. The explanation might be that a collection of pus in the ventricular system had become loculated and was thus not in communication with the subarachnoid space.

*Dr. O. C. Lloyd:* You say that the bacteriologists found several cells in this pus. Did they identify them?

*Mr. Hulme:* The report says "Many RBC and leucocytes. No organisms seen".

*Dr. Lloyd:* Yes, but did they identify these leucocytes?

*Mr. Hulme:* Apparently not.

*Professor Hewer:* Do you think they meant they were polymorphs?

*Mr. Hulme:* I don't know.

*Dr. Sandry:* I am pretty sure that, in fact, these were just degenerate cells.

*Dr. M. E. H. Halford:* You said that in the smear from the pus no organisms were seen. Did they include a Ziehl-Neelsen preparation for tubercle bacilli?

*Mr. Hulme:* I didn't discuss the actual techniques with the pathologists; they may well have done so.

*Dr. Sandry:* I am quite certain they did in this case.

*Professor Hewer:* It would have been a sad omission if they had not. When you said, Dr. Sandry, that you thought it might be an encephalitis, did you bear in mind that it appeared to be a unilateral lesion? I thought that might perhaps be a difficulty, if you use the word "encephalitis" as meaning a virus infection.

*Dr. Sandry:* I think that one could still entertain the diagnosis even if the physical signs were predominantly unilateral.

*Professor Hewer:* Does that shift in the ventricular system not mean that there was a unilateral lesion? If you had seen the shift in the ventricular system you would not have entertained the idea of encephalitis, would you?

*Dr. Sandry:* Yes, I think I would.

*Professor Hewer:* Of a virus encephalitis?

*Dr. Sandry:* Yes.

*Professor Hewer:* I find that surprising.

*Professor A. V. Neale:* Is Mr. Hulme quite clear that this was pus? It was not necrotic neoplastic tissue?

*Mr. Hulme:* Well, that is a difficult question. It was a creamy fluid.

*Professor Neale:* Is it ever possible to confuse necrotic neoplastic tissue with pus in the brain?

*Mr. Hulme:* Yes, very definitely. I have done it on more than one occasion.

*Professor Hewer:* It sounds as though speculation will not get us any further.

*Professor Neale:* May I ask one more question? Is it dangerous to do an air encephalogram with a lesion which is so deforming the ventricular system?

*Mr. Hulme:* Actually the degree of ventricular shift in this case, I think, was very slight. Furthermore she had no signs of raised intracranial pressure, the fundi were normal and the C.S.F. pressure at lumbar puncture was within the normal range, so we felt that it was reasonably safe to do this investigation.

*Professor Hewer:* Well, Dr. Sandry?

*Dr. Sandry:* Death was due to massive pulmonary embolism. This, however, while it was not exactly incidental, was not germane to the issue which is concerning us chiefly. I had better get off my chest straight away the fearful confession that I did not find the source of this massive embolism. There were traces of surgical assault appropriately situated on the scalp, but there was no other external abnormality; in fact the rest of the body outside the cranial cavity did not show any significant abnormality, so I think we may as well dismiss it straight away. The dura was a little tense when the skull was opened, but there was no subdural collection of blood or fluid and the venous sinuses were healthy. The brain itself showed fairly considerable swelling and flattening of the convolutions. The swelling was greater on the right than on the left, and was, I think, maximal in the right temporal and the right posterior parietal regions of the hemisphere. The superficial cerebral vessels were rather engorged. There was no evidence of a localized space-occupying lesion such as a tumour or abscess on external examination, other than the swelling in these two regions (which might, of course, have concealed something of that sort at greater depth). There was a possibility from the history—I must not put it any higher than that in view of the comments that have been made—there was a possibility of an encephalitis. The external appearances, shall I say, were not unlike those which might be expected in such a condition. So I took the unusual step, for me, of making a coronal slice through the unfixed brain, in order first of all to help to exclude a space-occupying lesion such as a tumour deep in the brain substance, and secondly to take specimens for virological examination.

I can show you the result of that first slice through the brain (Plate XVIII). The distortion of the brain is due to the fact that it was sliced when it was unfixed. Some swelling is evident, but it is not clear now, in this slice, which is the more swollen side. It looked when it was fresh as though it was a little more swollen on the right. It was evident that there was a curious softening and blurring of the pattern in the inferior frontal region and in the temporal pole on the right side, with a few tiny haemorrhages. At this stage I sent off material to Dr. Clarke at the virus laboratory at Canynge Hall, including a sample of the right temporal lobe and a piece of the medulla, taken with such precautions to ensure sterility as come naturally to a pathologist. I also sent some faecal matter and some blood. Perhaps Dr. Clarke could now tell us her findings.

*Dr. S. K. R. Clarke:* We emulsified the fragments that Dr. Sandry sent us in tissue culture medium and then inoculated them into various tissue cultures, including Hela cells, monkey kidney and human embryonic kidney. We grew no virus from the faeces or from the C.S.F and none from the medulla, but from the temporal lobe we isolated herpes simplex virus. That is, in the Hela cell tissue cultures inoculated with brain, after about nine days incubation, we saw the typical cytopathic effect you get with herpes. We confirmed that this was herpes simplex by showing that the infectivity of the virus was neutralized by a herpes anti-serum.

*Dr. Sandry:* In view of what we have already heard we must, of course, entertain the possibility that this was a contaminant! In the meantime, I completed the fixation and sectioning of the brain. I was able to confirm the swelling, softening and the loss of distinction between the grey and white matter especially in the posterior and inferior parts of the right temporal lobe and in the inferior frontal cortex which I have already described. There were some tiny haemorrhages in the right frontal lobe, mostly in the grey matter, and in the right occipital lobe there were some slightly less delicate haemorrhages which I think were in the region through which the ventricular tap had been performed. In the mid-brain and pons, especially the latter, there were further small haemorrhages.

By this time the report on the virus study was to hand, so before embarking upon the histological examination I knew what Dr. Clarke at any rate wanted me to look for. I find this a very great help, particularly in such cases as this, which to me seem very complex. The changes already seen with the naked eye, apart from the asymmetry, did in fact accord reasonably well with those described in herpes simplex encephalitis. Such softening, mostly in the cortex, patchy in distribution and tending to affect the temporal and frontal lobes rather than other parts of the brain, has frequently been described in this condition. The mid-brain haemorrhages perhaps did not fit in quite so well, but they also have figured in one or two reports. I therefore took sections from various parts of the cerebrum and from the mid-brain, pons and cerebellum. I am afraid I did not embark upon a full survey of the brain in the manner of a true neuropathologist. The changes which I found can be summarized fairly easily.

PLATE XVIII

*Coronal section of brain, showing softening and haemorrhages in right temporal pole and both inferior frontal regions.*

PLATE XIX

*Mounted section from inferior frontal region, showing disintegration of grey matter.*

× 4·3

PLATE XX

*An intranuclear inclusion body.*
× 1,650

The leptomeninges showed a mononuclear cellular infiltration, chiefly lymphocytic, with some histiocytes and an occasional plasma cell. This is the sort of non-specific cellular infiltration which one would expect in view of the findings in the C.S.F. during life. Sections taken through the inferior frontal region of the brain showed relatively little change in the white matter, but extensive and gross disintegration of the grey matter. The photograph (Plate XIX) gives you some idea of the severity of this disorganization. In other parts of the cortex, less severe damage was seen. In these damaged areas the small blood vessels showed lymphocytic cuffing, and there was damage to nerve cells ranging from slight swelling to complete destruction. In the most severely damaged areas there remained no trace of the normal cortical structure. Various types of inflammatory cells were present, including lymphocytes, plasma cells and so-called compound granular corpuscles, laden with fat liberated by the break-down of myelin; the actual destruction of tissue was, however, a far more striking feature than the inflammatory infiltration, which indeed appeared rather slight for such a devastating lesion.

So much for the cerebral cortex. I did not explore the basal ganglia very fully, but they appeared to be relatively slightly affected. The cerebellum also showed only slight changes; lymphocytic cuffing of vessels was present, but the pattern of the tissue was not altered and the damage to nerve cells appeared to be slight.

The distribution of these lesions was on the whole similar to that which has been described in cases of encephalitis due to the virus of herpes simplex, and the histological appearances are also similar to those which have been seen in such cases. There remains one other feature: in this type of encephalitis many observers have been able to demonstrate intra-nuclear inclusion bodies in certain cells of the brain. I will not weary you with details of my long and frustrating search for these. While taking lunch during an interval I met a distinguished and, indeed, eminent colleague, a man whom I greatly respect. I was little encouraged when, on hearing of my difficulties, he remarked—"If your case is anything like the one I was shown some time ago, it would be difficult for a blind man to miss the inclusion bodies!" Stung perhaps by this, I did then begin to have some success, and eventually, with the aid of optical equipment of a higher quality than I usually use, I managed to convince myself and my bacteriological colleague that intra-nuclear inclusion bodies were indeed present. They were pinkish in colour, rather ill-defined, and lay usually in swollen nuclei in which the nuclear chromatin tended to be scattered around the periphery of the nucleus. Photographed in monochrome (Plate XX) they closely resemble nucleoli, but under the microscope they were quite clearly recognizable as inclusion bodies. It is difficult to identify the cells in which they lie since these have evidently suffered damage and distortion, but some of them are undoubtedly oligodendrocytes; others may be nerve cells.

The lesions which had been noted with the naked eye in the pons and mid-brain proved histologically to be little areas of early haemorrhagic softening. I believe that these were due, not directly to the virus, but to ischaemia following downward displacement of the mid-brain. Such lesions are commonly found in cases in which there has been a rise in the supra-tentorial pressure, and in this case the degree of oedema of the cerebrum was quite sufficient to account for them.

To sum up, I think we can claim that in this case the clinical history, the lesions in the brain (both in their distribution and in their histological characteristics) and, most important, the results of the virus studies, all point to a diagnosis of encephalitis due to the virus of herpes simplex. I hope that others may be able to tell us why this ordinarily not very important organism should occasionally behave in this very virulent fashion.

*Professor Hewer:* Have any of our virological friends anything to tell us about the role of herpes simplex in encephalitis?

*Dr. Clarke:* I think it is always a manifestation of primary infection with the virus, never of secondary, recurrent herpes simplex. Primary infection, of course, happens in children, rarely in adults. About 85 per cent of children get infected with herpes by the age of 15. A few people do become infected for the first time as adults, though for some reason this seems to be rather rare, so it is possible that this patient got to the age of 57 before she got a primary infection with the virus. There is some evidence that herpes is possibly rather like polio, and that the older you are when you get your primary infection, the more likely you are to get a severe attack.

*Dr. Lloyd:* Does that mean that people who commonly get skin lesions of herpes simplex are very unlikely to get encephalitis?

*Dr. Clarke:* They could have got encephalitis when they got their primary infection as children, but not when they got recurrent herpes labialis.

*Professor Hewer:* Yes, by the time one has got to the stage of having recurrent herpes one is all right as far as the brain is concerned.

Are there any figures available of the antibody titre against herpes simplex in the general population?

*Dr. Clarke:* I think so. One fairly recent survey showed that something like 85 per cent of children had antibodies by the age of 15 and that after that age the percentage with antibodies increased only very slightly with age.

*Professor Hewer:* I suppose it is possible that it depends on the portal of entry whether you are going to get encephalitis or not. When a virus enters through the skin it is often less dangerous than by inhalation of droplets. Has Dr. Clarke any information on this in the case of herpes simplex?

*Dr. Clarke:* I think in a lot of cases of herpes encephalitis there is a history of stomatitis or some other manifestation of primary infection at the beginning of the illness.

*Dr. Sandry:* In one or two of the cases in infants there have been severe herpetic lesions on the lips and skin of the face.

*Professor Hewer:* And encephalitis followed?

*Dr. Sandry:* Yes, encephalitis followed or appeared simultaneously.

*Dr. Macrae:* We have had a number of cases of stomatitis in infants and Dr. Clarke consistently grows herpes simplex virus from the patients' mouths. One assumes that children with stomatitis have a primary infection of herpes simplex.

*Dr. H. R. Cayton:* It would be possible to discover whether this woman in fact suffered from recurrent herpes.

*Professor Hewer:* From the history?

*Dr. Cayton:* Her husband would know.

*Dr. Macrae:* When I saw her husband everything was in chaos. She had just been moved to hospital and even he was a little bit confused. We were not thinking of herpes simplex virus infection.

*Professor Hewer:* After that provocative remark of mine about the asymmetrical character of the lesion, would Dr. Sandry like to say anything about the distribution of the lesions? Were you at all surprised to see swelling more on one side than the other?

*Dr. Sandry:* I think that this is sometimes a feature of this type of encephalitis. The distribution of the lesions is not at all uniform, and one lobe, or one part of one lobe, is not uncommonly more severely affected than another. This appears to be true especially of the temporal and inferior frontal regions. I do not know if there is any significance in the fact that these areas are more likely to be involved severely.

*Professor Hewer:* Presumably the portal of entry has something to do with it.

*Dr. Sandry:* Yes, that might be so. The asymmetry of the lesions in this case was certainly a striking feature clinically.

*Professor Hewer:* The lesions produced by specific viruses in the central nervous system often have a distinctive appearance, and in most cases they are symmetrical.

Has Dr. Norman any comment on that?

*Dr. R. M. Norman:* I have been reading the same book on this subject as has Dr. Sandry.

*Dr. Sandry:* Dr. Norman lent it to me, but he made sure that he got it back by last Saturday!

*Dr. Norman:* From the findings so modestly presented by Dr. Sandry, a clear picture emerges of what is called "necrotising encephalitis", a group of conditions of which herpes simplex encephalitis is one member. The necrosis can be seen by the naked eye and typically affects the temporal lobes and the orbital surface of the frontal lobes. In some of these encephalitides inclusion bodies are not to be found, but the only one in which virus has so far been recovered is the herpes simplex type. It may be difficult to decide whether the necrosis is ischaemic in origin, but it has been said that if the necrosis affects the molecular layer as well as the underlying grey matter, this is a point in favour of it being due to encephalitis. I think we saw this in the present case. On the other hand, I believe the central pontine lesions may well be due to compression of vessels following displacement of the brain stem by the inflammatory swelling of the brain. I take it there was no infiltration of the vessels in these areas.

*Dr. Sandry:* There was perivascular lymphocytic cuffing here and there in the pons, but this is often very much more widespread than are the actual cellular lesions caused by the virus.

*Dr. A. C. Hunt:* Was the necrosis of the cortex of such a degree that this would account for the "pus"?

*Dr. Sandry:* I think so, yes. I am quite sure this was not, in fact, pus; I could find no trace of pus in any part of the brain, whereas there was certainly a good deal of softened and degenerate brain tissue.

*Question:* Is this virus disseminated around the body inside the leucocytes or lymphocytes, as we know the polio virus can be disseminated?

*Dr. D. B. Peacock:* I think the spread is from cell to cell.

*Professor Hewer:* Will Dr. Norman return to this question of the recognition of herpes encephalitis? You say that there are cases in which herpes simplex virus is not obtained. Is that possibly because they were not adequately examined? I believe it needs some skill and a negative result does not necessarily mean that the virus was not present.

*Dr. Norman:* I could not give an opinion. Certainly it would be advisable to send specimens to Dr. Clarke. I have always heard that in the true herpes simplex variety the number of inclusion bodies is pretty large and you ought to be able to find them fairly easily. If a careful search reveals none I should have thought it was against the case being one of herpes simplex encephalitis. But that is only the result of my week-end reading.

*Dr. Sandry:* There is no longer any secret about the identity of my lunch hour companion!

*Professor Hewer:* Well, thank you all very much.

## Figures and Tables

**Figure f1:**
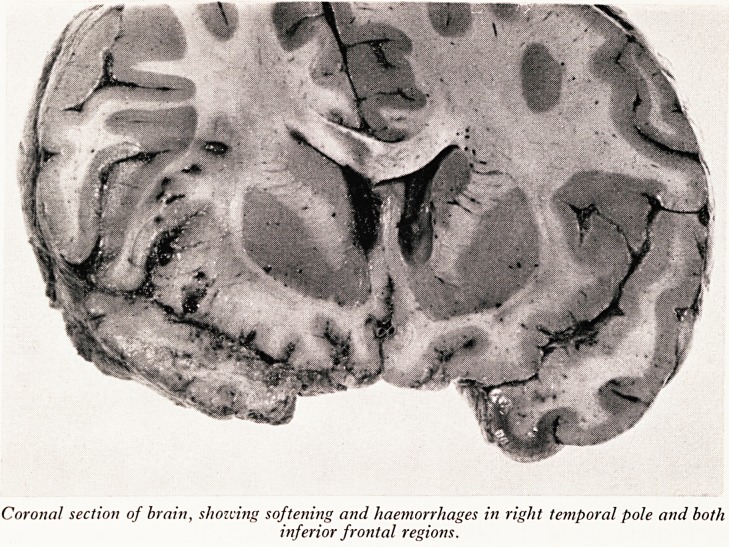


**Figure f2:**
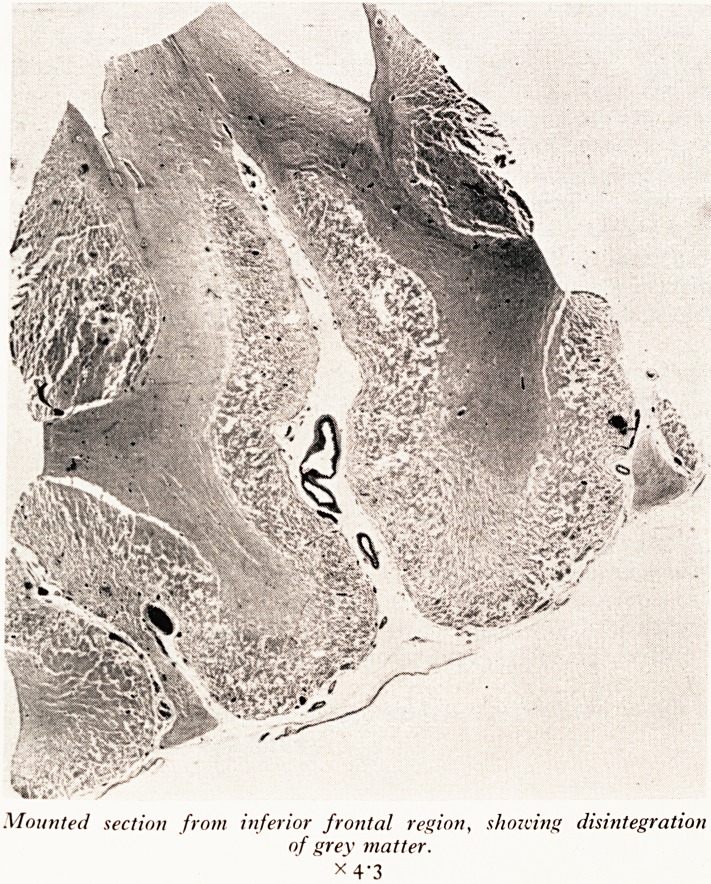


**Figure f3:**